# Effects of spironolactone on exercise blood pressure in patients at increased risk of developing heart failure: report from the HOMAGE trial

**DOI:** 10.1038/s41440-024-01843-z

**Published:** 2024-09-06

**Authors:** Fang-Fei Wei, Pierpaolo Pellicori, João Pedro Ferreira, Arantxa González, Beatrice Mariottoni, De-Wei An, Job A. J. Verdonschot, Chen Liu, Fozia Z. Ahmed, Johannes Petutschnigg, Patrick Rossignol, Stephane Heymans, Joe Cuthbert, Nicolas Girerd, Andrew L. Clark, Yan Li, Tim S. Nawrot, Javier Díez, Faiez Zannad, John G. F. Cleland, Jan A. Staessen, on behalf of the HOMAGE Investigators, Arantxa González, Chen Liu, Fozia Z. Ahmed, Joe Cuthbert, Andrew L. Clark, Kei Asayama, Erwan Bozec, Hans P. Brunner La Rocca, Franco Cosmi, John G. F. Cleland, Tim Collier, Javier Díez, Frank Edelmann, João P. Ferreira, Nicolas Girerd, Stephanie Grojean, Mark Hazebroek, Stephane Heymans, Tine W. Hansen, Javed Khan, Begoñia López, Roberto Latini, Beatrice Mariottoni, Ken McDonald, Gladys E. Maestre, María U. Moreno, Mamas A. Mamas, Anne Pizard, Burkert Pieske, Johannes Petutschnigg, Pierpaolo Pellicori, Patrick Rossignol, Philippe Rouet, Suzanna Ravassa, Jan A. Staessen, Lutgarde Thijs, Job A. J. Verdonschot, Fang-Fei Wei, Faiez Zannad

**Affiliations:** 1 Department of Cardiology, The First Affiliated Hospital of Sun Yat-Sen University, Guangzhou Guangdong, China; 2 Non-Profit Research Association Alliance for the Promotion of Preventive Medicine (APPREMED), Mechelen, Belgium; 3 Robertson Centre for Biostatistics, Institute of Health and Wellbeing, University of Glasgow, Glasgow Royal Infirmary, Glasgow, UK; 4 Department of Physiology and Cardiothoracic Surgery, Faculty of Medicine, University of Porto Portugal, Porto, Portugal; 5 Portugal Heart Failure Clinics, Department of Internal Medicine, Centro Hospitalar de Vila Nova de Gaia/Espinho, Vila Nova de Gaia, Portugal; 6 Program of Cardiovascular Diseases, CIMA. Universidad de Navarra and IdiSNA, Pamplona, Spain CIBERCV, Carlos III Institute of Health, Madrid, Spain; 7 Department of Cardiology, Cortona Hospital, Arezzo, Italy; 8 Department of Cardiovascular Medicine, Shanghai Key Laboratory of Hypertension, Shanghai Institute of Hypertension, State Key Laboratory of Medical Genomics, National Research Centre for Translational Medicine, Ruijin Hospital, Shanghai Jiaotong University School of Medicine, Shanghai, China; 9 Department of Cardiology, Maastricht University Medical Centre, Maastricht, The Netherlands; 10 Division of Cardiovascular Sciences, School of Medical Sciences, Faculty of Biology, Medicine and Health, Manchester Academic Health Science Centre, University of Manchester, Manchester, UK; 11 Department of Internal Medicine and Cardiology, Campus Virchow Klinikum, Charité University Medicine Berlin, Berlin Institute of Health and German Center for Cardiovascular Research, Partner Site Berlin, Berlin, Germany; 12 Université de Lorraine, Inserm, Centre d’Investigation Clinique Plurithématique 1433, U1116, CHRU de Nancy, F-CRIN INI-CRCT, Nancy, France; 13 Department of Cardiology, University of Hull, Castle Hill Hospital, Cottingham, East Riding of Yorkshire, Hull, UK; 14 Research Unit Environment and Health, KU Leuven Department of Public Health and Primary Care, University of Leuven, Leuven, Belgium; 15 Centre for Environmental Sciences, Hasselt University, Hasselt, Belgium; 16 Biomedical Science Group, University of Leuven, Leuven, Belgium; 17 Department of Medical Statistics, London School of Hygiene and Tropical Medicine, London, UK; 18 Fondation Force, Research and Consulting Department, EDDH, Centre de Médecine Préventive, Vandoeuvre les Nancy, Nancy, France; 19 Department of Cardiovascular Medicine, Istituto di Ricerche Farmacologiche Mario Negri—IRCCS, Milan, Italy; 20 St. Vincent’s University Healthcare Group, and School of Medicine, University College Dublin, Dublin, Ireland; 21 Centre for Prognosis Research, Institute for Primary Care and Health Sciences, Keele University, Newcastle, UK; 22 Equipe obésité et insuffisance cardiaque, Université Paul Sabatier, Inserm, Toulouse, France; 23 Studies Coordinating Centre, Research Unit Hypertension and Cardiovascular Epidemiology, Department of Cardiovascular Sciences, University of Leuven, Leuven, Belgium

**Keywords:** Coronary heart disease, Exercise capacity, Incremental shuttle walk test, Heart failure, Spironolactone

## Abstract

None of the spironolactone trials in heart failure (HF) assessed the blood pressure (BP) responses to exercise, while conflicting results were reported for exercise capacity. In the HOMAGE trial, 527 patients at increased HF risk were randomized to usual treatment with or without spironolactone (25–50 mg/day). The current substudy included 113 controls and 114 patients assigned spironolactone, who all completed the incremental shuttle walk test at baseline and months 1 and 9. Quality of life (QoL) was assessed by EQ5D questionnaire. Between-group differences (spironolactone minus control [Δs]) were analyzed by repeated measures ANOVA with adjustment for baseline and, if appropriate, additionally for sex, age and body mass index. Δs in the pre-exercise systolic/diastolic BP were −8.00 mm Hg (95% CI, −11.6 to −4.43)/−0.85 mm Hg (−2.96 to 1.26) at month 1 and −9.58 mm Hg (−14.0 to −5.19)/−3.84 mm Hg (−6.22 to −1.47) at month 9. Δs in the post-exercise systolic/diastolic BP were −8.08 mm Hg (−14.2 to −2.01)/−2.07 mm Hg (−5.79 to 1.65) and −13.3 mm Hg (−19.9 to −6.75)/−4.62 mm Hg (−8.07 to −1.17), respectively. For completed shuttles, Δs at months 1 and 9 were 2.15 (−0.10 to 4.40) and 2.49 (−0.79 to 5.67), respectively. Δs in QoL were not significant. The correlations between the exercise-induced BP increases and the number of completed shuttles were similar in both groups. In conclusion, in patients at increased risk of developing HF, spironolactone reduced the pre- and post-exercise BP, but did not improve exercise capacity or QoL.

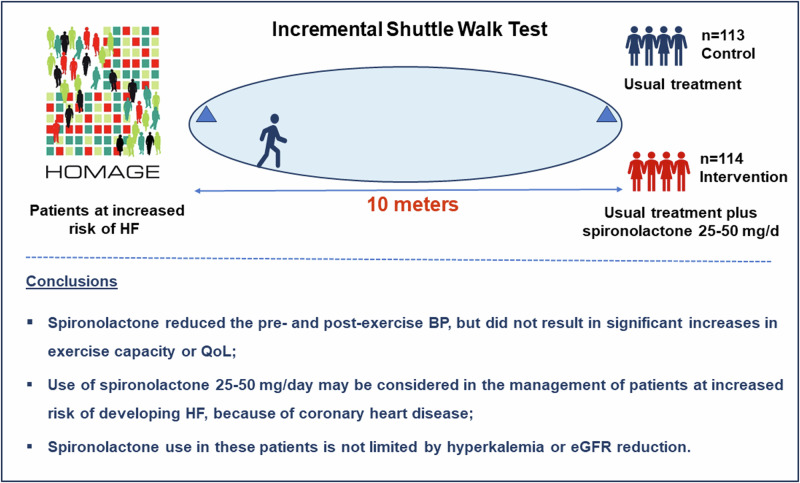

## Introduction

Exercise capacity provides important diagnostic and prognostic information in patients with chronic cardiac [1–3] and pulmonary conditions [4]. Exercise tests are widely used to evaluate the efficacy of new therapies and to assess changes in heart rate and blood pressure (BP) in response to exercise [5–7]. Incremental cardiopulmonary exercise testing with metabolic gas exchange measurement (CPET) is the gold standard method to assess maximal aerobic capacity [8]. However, routine CPET is costly and challenging. The incremental shuttle walk test (ISWT) is a symptom-limited maximal test and can track changes in the functional capacity of patients with chronic heart failure (HF) [9–12]. Exercise capacity as measured by the ISWT correlates closely with the results of CPET [13]. The correlations between distance covered during the ISWT and peak oxygen consumption range between 0.67 and 0.95 [13]. Trials have shown that mineralocorticoid receptor inhibition with spironolactone [14–18], eplerenone [19], finerenone [20], or spironolactone on top sodium-glucose co-transporter 2 inhibitors [21] improves outcomes in HF. However, few trials [15–18] have assessed the functional effect of spironolactone and none has reported on the BP responses to exercise.

We analyzed data from the HOMAGE (Heart OMics in Aging) trial, a prospective randomized trial in patients at increased risk of developing HF [22]. HOMAGE evaluated the effect of spironolactone on biomarkers of fibrosis and cardiac remodeling [23]. In this prespecified secondary analysis, we investigated the influence of spironolactone on the BP responses to exercise and exercise capacity. We also investigated the correlation between the changes from baseline to the last follow-up visit in the pre- and post-exercise BP and the corresponding changes in circulating biomarkers, echocardiographic measurements, and quality of life (QoL).

## Methods

### Study participants

**Fig. 1:**
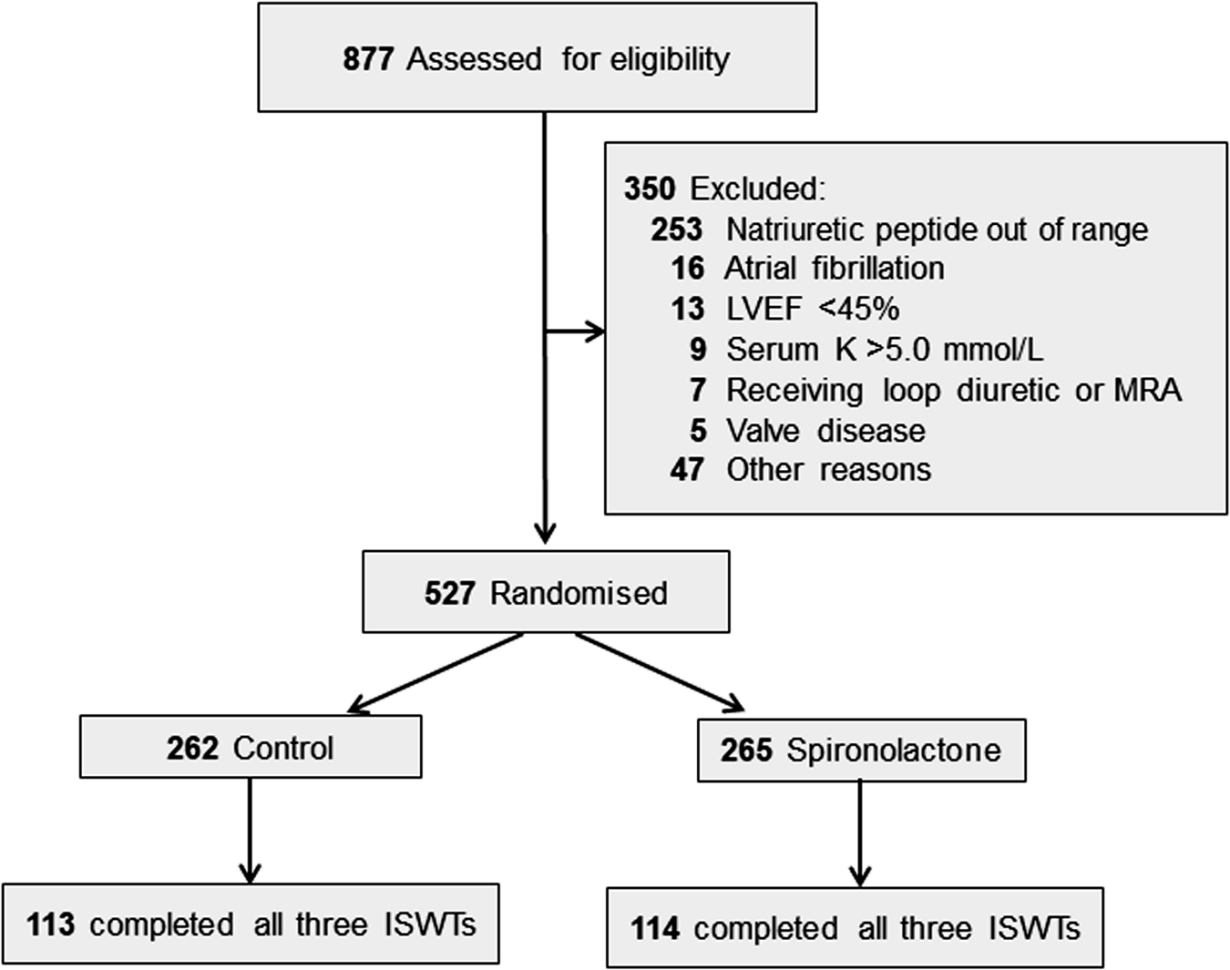
Consort diagram showing patient disposition, including screening, randomization and follow-up, and selection of patients for the current analysis. Analyses include 227 patients who completed the ISWT at months 0, 1, and 9. LVEF left ventricular ejection fraction; MRA mineralocorticoid receptor antagonism

HOMAGE is a multicenter open-label trial with blinded endpoint evaluation (registration number: NCT02556450) [23], conducted in nine centers in the United Kingdom, France, Italy, Ireland, Germany, and the Netherlands. Each center had its own recruitment strategies. The protocol was approved by the Greater Manchester Central Research Ethics Committee (reference, 16/NW/0012; EudraCT number: 2015-000413-48) as well as by each center’s local Ethics Committee. Patients of either sex, aged ≥60 years were eligible, provided that they were at increased risk of developing HF because they already had or were likely to develop coronary heart disease. Additionally, eligible patients had to have a serum N-terminal pro B-type natriuretic peptide (NT-proBNP) of 125–1000 ng/L or a serum BNP of 35–280 ng/L. These ranges excluded patients at low HF risk as well as those with advanced disease requiring further investigation and treatment. Of 877 screened patients (Fig. 1), 527 were randomized to spironolactone 25–50 mg per day (n = 265) on top of usual treatment or usual treatment alone (n = 262) [23]. Of all patients randomized and followed-up in the HOMAGE trial, 450/527 (85.4%), 324/516 (62.8%), and 400/506 (79.1%) completed the ISWT at baseline and at months 1 and 9. The current analyses includes 227 patients who completed the ISWTs at each of these time points, the justification being that evaluating the same patients at each time point increases the comparability of the data over time. Of the 227 patients, 113 were randomized to control and 114 to spironolactone.

### Measurements

Exercise capacity was measured by ISWT [24], as described in detail in the online Supplementary Information. Skilled personnel conducted the ISWT around two cones, 9 m apart [22]. Patients walk in a circle around the cones (“the shuttle”), which adds 1 meter to the distance covered. The walking speed is determined by bleeps played from a compact disc. After every minute, walking speed increased. There are up to 12 levels of speed, and potentially 102 shuttles. BP was measured at rest, immediately after the ISWT, and 1, 2, 3, and 5 min later. The ISWT was performed at baseline and at the 1- and 9-month visits. Trained observers recorded BP by conventional sphygmomanometry by auscultation of the Korotkoff sounds. Heart rate and the Borg dyspnea score were recorded pre-exercise and immediately post-exercise. Serum was analyzed for circulating fibrosis markers (Supplementary Fig. 1), high-sensitivity troponin T (hsTnT), and NT-proBNP, as described elsewhere [23, 25]. Commercial radio-immunoassays (Orion Diagnostica, Espoo, Finland) were used to measure serum collagen type-1 C-terminal telopeptide (CITP). Serum procollagen type I C-terminal propeptide (PICP), a marker of collagen type I synthesis was measured using the METRA EIA kit (Quidel Corporation, San Diego, CA). The lower limit of detection was 0.6 ng/mL for CITP and 0.2 ng/mL for PICP. All inter- and intra-assay coefficients of variation were <10%. Glomerular filtration rate was estimated (eGFR) from serum creatinine by the Chronic Kidney Disease Epidemiology Collaboration (CKD-EPI) formula [26]. QoL was assessed using the EQ5D visual analog score (https://euroqol.org/eq-5d-instruments). Echocardiography was performed according to the guidelines of the American Society of Echocardiography [27]. The echocardiographic traits of interest include the ratio of the peak early diastolic transmitral flow to the peak of early diastolic mitral annular movement (E/e’) as index of the left ventricular (LV) filling pressure, left atrial volume (LAVI), and left ventricular mass (LVMI), both indexed to body surface area. Patients completed questionnaires to report side effects at month1 and 9.

### Statistical analysis

For database management and statistical analysis, SAS software, version 9.4 (SAS Institute Inc., Cary, NC) was used. Deviation from the normal distribution was assessed by the Shapiro-Wilk statistic. For comparison of means, we used a t-test or Wilcoxon-Mann-Whitney test depending on the distribution. For comparison of proportions, we applied the χ2-statistic. Pearson correlation coefficients between variables of interest were computed and compared between groups by the Fisher z-transform [28]. Significance was a 2-sided α-level of ≤0.05. Prior to analysis, PICP, CITP, and NT-proBNP were logarithmically transformed (base 10) to approximate the normal distribution. The between-group differences in continuous measurements at months 1 and 9 were calculated by subtracting the mean changes from baseline in the control group from the corresponding changes in the spironolactone group always with adjustment for baseline and additionally, if so mentioned, also for sex, age, and body mass index. For logarithmically transformed variables, within-group changes from baseline to the 1- and 9-month visits and between-group difference were expressed as ratios by multiplying the antilog of the difference between two logarithmically transformed values by 100 to convert results to the percentage scale. Significance of the between-group differences in the serial BP values during the ISWT was analyzed by repeated measures ANOVA without and with adjustment for sex, age, and body mass index. If the baseline QoL data were missing, they were replaced with the 1-month scores, because there were no differences between baseline and the 1-month QoL scores in the 124 patients for whom data were available at both time points. In sensitivity analyses, the data were stratified for sex and the medians of age, LV ejection fraction (LVEF), and eGFR.

## Results

### Patient characteristics

Descriptive data for the 227 patients are shown in Table 1. The patients were intensively treated with antihypertensive agents (n = 163; 71.8%) and lipid-lowering drugs (n = 203; 89.4%), mainly statins (n = 197; 86.8%), antiplatelet drugs (n = 168; 74.0%), and antidiabetic agents (n = 81; 35.7%). There were no significant differences between the patients randomized to placebo or spironolactone. At baseline (Table 2), exercise capacity measured by the number of completed shuttles and systolic and diastolic BP both before and after exercise were similar in both groups (*P* ≥ 0.11). The 227 patients included in the substudy had broadly similar characteristics compared to the 300 HOMAGE patients not included (Supplementary Table 1). However, the patients reported here were younger, had a higher eGFR, were less likely to smoke (5.73% versus 10.3%; *P* = 0.040), and had a higher prevalence of ischemic heart disease (79.3% versus 66.3%; *P* = 0.001).

**Table 1:** Baseline characteristics of patients by trial arm

Characteristic	Control	Spironolactone	*P* value
Number with characteristic	113	114	
Women	26 (23.0)	23 (20.2)	0.60
Caucasian	112 (99.1)	109 (96.5)	0.50
Current smoking	8 (7.08)	5 (4.39)	0.56
Hypertension	84 (74.3)	87 (76.3)	0.73
Treated hypertension	81 (96.4)	82 (94.3)	0.97
Diabetes	45 (39.8)	42 (36.8)	0.64
Treated diabetes	40 (88.9)	41 (97.6)	0.93
History of coronary artery disease	90 (79.7)	90 (79.0)	0.90
History of myocardial infarction	46 (51.1)	47 (52.2)	0.88
Clinical characteristics			
Age (years)	72.4 ± 5.93	72.0 ± 6.16	0.61
BMI (kg/m^2^)	28.7 ± 4.96	29.6 ± 5.42	0.18
Waist-to-hip ratio	0.97 ± 0.07	0.98 ± 0.07	0.15
Biochemistry			
Serum sodium (mmol/L)	139 (138–141)	138 (136–139)	0.40
Serum potassium (mmol/L)	4.3 (4.1–4.6)	4.5 (4.2–4.7)	0.42
eGFR (mL/min/1.73 m^2^)	72 (61–82)	76 (61–89)	0.074
hsTNT (ng/L)	12.2 (8.7–17.4)	11.7 (8.4–15.0)	0.064
Plasma NT-proBNP (ng/L)	204 (118–289)	170 (120–331)	0.61

Data are presented as arithmetic mean ± SD, median (IQR), or n (%)

*hsTNT* high-sensitivity troponin-T, *NT-proBNP* N-terminal pro B-type natriuretic peptide, *eGFR* glomerular filtration rate estimated from serum creatinine according to the Chronic Kidney Disease Epidemiology equation

**Table 2:** Blood pressure and endurance at randomization and at 1 and 9 months by treatment group

Variable	Control (n = 113)	Spironolactone (n = 114)	Difference (95% CI)	*P* Value
Pre-exercise BP				
Systolic, mm Hg				
Baseline	143.5 ± 20.4	139.0 ± 21.4	−4.49 (−9.95 to 0.98)	0.11
Change at 1 month	−4.86 ± 1.41^‡^	−12.9 ± 1.42^‡^	−8.00 (−11.6 to −4.43)	<0.001
Change at 9 months	−5.13 ± 1.74^†^	−14.7 ± 1.76^‡^	−9.58 (−14.0 to −5.19)	<0.001
Diastolic, mm Hg				
Baseline	77.1 ± 11.4	75.6 ± 10.5	−1.53 (−4.39 to 1.34)	0.29
Change at 1 month	−2.44 ± 0.84^†^	−3.29 ± 0.85^‡^	−0.85 (−2.96 to 1.26)	0.43
Change at 9 months	−1.47 ± 0.94	−5.32 ± 0.95^‡^	−3.84 (−6.22 to −1.47)	0.002
Post-exercise BP				
Systolic, mm Hg				
Baseline	172.3 ± 29.0	171.2 ± 31.0	−1.14 (−9.01 to 6.72)	0.77
Change at 1 month	−4.01 ± 2.41	−12.1 ± 2.44^‡^	−8.08 (−14.2 to −2.01)	0.009
Change at 9 months	−3.86 ± 2.61	−17.2 ± 2.64^‡^	−13.3 (−19.9 to −6.75)	<0.001
Diastolic, mm Hg				
Baseline	81.3 ± 13.4	82.5 ± 17.6	1.23 (−2.87 to 5.32)	0.56
Change at 1 month	−0.57 ± 1.48	−2.64 ± 1.49	−2.07 (−5.79 to 1.65)	0.27
Change at 9 months	−1.46 ± 1.37	−6.08 ± 1.38^‡^	−4.62 (−8.07 to −1.17)	0.009
All time points				
Systolic, mm Hg				
Baseline	157.9 ± 21.3	155.1 ± 22.9	−2.82 (−8.61 to 2.98)	0.34
Change at 1 month	−4.51 ± 1.56^†^	−12.4 ± 1.58^‡^	−7.86 (−11.8 to −3.92)	<0.001
Change at 9 months	−4.64 ± 1.87*	−15.9 ± 1.89^‡^	−11.2 (−16.0 to −6.52)	<0.001
Diastolic, mm Hg				
Baseline	79.2 ± 10.7	79.0 ± 11.8	−0.15 (−3.10 to 2.80)	0.92
Change at 1 month	−1.52 ± 0.94	−2.90 ± 0.96^†^	−1.39 (−3.77 to 1.00)	0.25
Change at 9 months	−1.43 ± 0.95	−5.69 ± 0.96^‡^	−4.25 (−6.64 to −1.86)	0.001
∆BP from rest to exercise				
Systolic, mm Hg				
Baseline	28.8 ± 26.3	32.2 ± 27.2	3.34 (−3.66 to 10.3)	0.35
Change at 1 month	0.34 ± 2.40	0.52 ± 2.41	0.18 (−5.84 to 6.20)	0.95
Change at 9 months	1.05 ± 2.39	−2.79 ± 2.41	−3.84 (−9.85 to 2.17)	0.21
Diastolic, mm Hg				
Baseline	4.14 ± 12.6	6.89 ± 16.9	2.75 (−1.14 to 6.64)	0.16
Change at 1 month	1.72 ± 1.46	0.75 ± 1.48	−0.97 (−4.67 to 2.73)	0.60
Change at 9 months	−0.19 ± 1.36	−0.51 ± 1.37	−0.32 (−3.75 to 3.11)	0.85
Completed shuttles, n				
Baseline	48.5 ± 22.1	46.4 ± 21.7	−2.11 (−7.84 to 3.63)	0.47
Change at 1 month	−0.71 ± 0.89	1.44 ± 0.91	2.15 (−0.10 to 4.40)	0.061
Change at 9 months	0.26 ± 1.29	2.71 ± 1.31*	2.49 (−0.75 to 5.74)	0.13

Pre- and post-exercise blood pressure (BP) refers to the blood pressure measured immediately before and after the incremental shuttle walk test. Mean between-group differences (spironolactone minus control level) are given with 95% confidence interval and the significance level. Changes are adjusted for the baseline value, sex, age, and body mass index. ΔBP indicates the increase in BP from rest to immediately post-exercise. Significance of the within-group changes: *P ≤ 0.05, ^†^*P* ≤ 0.01, and ^‡^*P* ≤ 0.001

### Pre-exercise BP

**Fig. 2:**
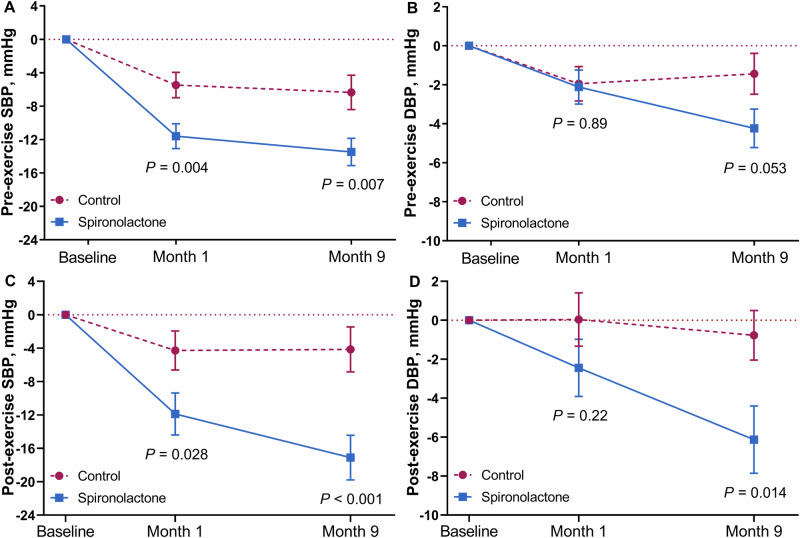
Changes from baseline to months 1 and 9 in the pre-exercise (**A**, **B**) and post-exercise (**C**, **D**) systolic (**A**, **C**) and diastolic (**B**, **D**) BP by randomization group. Data points are unadjusted mean changes ± SE. *P* values are for the comparison between intervention and control

Although systolic BP fell between the baseline and the 1-month visit in the placebo group, the fall was much greater in the spironolactone group (Fig. 2). Compared with the control group, the 9-month pre-exercise systolic BP remained significantly reduced in the spironolactone arm (−6.34 versus −13.5 mm Hg; 95% CI, −10.4 to −2.25 versus −16.7 to −10.2 mm Hg; *P-*value for the between-group difference: 0.007). These results were unaffected by sex (Supplementary Fig. 2), age (Supplementary Fig. 3), LVEF (Supplementary Fig. 4), or eGFR (Supplementary Fig. 5). In analyses, adjusted for sex, age, body mass index, and the baseline systolic BP level (Table 2), the resting pre-exercise systolic BP at 1 and 9 months decreased 8.00 mm Hg and 9.58 mm Hg more in the spironolactone than in the control group. For diastolic BP, with similar adjustments applied (Table 2), the between-group difference was not significant at 1 month (spironolactone minus control group: −0.85 mm Hg; *P* = 0.43), but was significant at 9 months (−3.84 mm Hg; *P* = 0.002).

### Post-exercise BP

Figure 2 shows the systolic and diastolic BP responses to ISWT. In unadjusted analyses, at 1 month, systolic/diastolic BP, measured immediately post-exercise, did not change in the control group (−4.27/0.04 mm Hg; 95% CI, −8.90 to 0.35/−2.68 to 2.75 mm Hg; *P* ≥ 0.070), whereas in the spironolactone group at month 1 the post-exercise systolic BP (−11.9 mm Hg; 95%CI, −16.9 to −6.90 mm Hg; *P* < 0.001), but not post-exercise diastolic BP (−2.45 mm Hg; 95%CI, −5.37 to 0.47 mm Hg; *P* = 0.10) decreased. The 9-month post-exercise BP did not change compared to baseline in the control group (−4.15/−0.77 mm Hg; 95% CI, −9.49 to 1.19/−3.30 to 1.76 mm Hg; *P* ≥ 0.13), but remained reduced on spironolactone (−17.1/−6.13 mm Hg; 95% CI, −22.4 to −11.8/−9.56 to −2.70 mm Hg; *P* < 0.001). In analyses adjusted for sex, age, BMI, and the baseline BP level (Table 2), the post-exercise systolic BP at months 1 and 9 decreased 8.08 mm Hg and 13.3 mm Hg more in the spironolactone than in the control group. For diastolic BP with similar adjustments applied (Table 2), the between-group difference in the post-exercise diastolic BP was not significant at 1 month (spironolactone minus control group: −2.07 mm Hg; *P* = 0.27), but was significant at 9 months (−4.62 mm Hg; *P* = 0.009). Compared to placebo, spironolactone did not affect the increase in BP in response to ISWT (Table 2), pre- and post-exercise heart rate, or Borg score (Table 3).

**Table 3:** Other ISWT parameters at randomization and at 1 and 9 months by treatment group

Variable	Control (n = 113)	Spironolactone (n = 114)	Difference (95% CI)	*P* Value
Pre-exercise HR, bpm				
Baseline	62.1 ± 9.46	62.0 ± 9.79	−0.09 (−2.61 to 2.43)	0.94
Change at 1 month	−0.94 ± 0.74	0.16 ± 0.75	−1.10 (−0.76 to 2.96)	0.24
Change at 9 months	−0.39 ± 0.90	−1.13 ± 0.91	−0.74 (−3.00 to 1.52)	0.52
Post-exercise HR, bpm				
Baseline	83.1 ± 21.1	81.2 ± 20.1	−1.95 (−7.34 to 3.44)	0.48
Change at 1 month	−0.90 ± 1.32	0.73 ± 1.34	1.63 (−1.71 to 4.98)	0.34
Change at 9 months	−2.88 ± 1.53	−1.60 ± 1.55	1.28 (−2.58 to 5.14)	0.52
Borg scale, unit				
Baseline	4.21 ± 1.80	3.88 ± 1.75	−0.34 (−0.80 to 0.13)	0.16
Change at 1 month	0.32 ± 0.17	0.24 ± 0.17	0.08 (−0.51 to 0.35)	0.72
Change at 9 months	0.39 ± 0.19*	0.50 ± 0.20*	0.11 (−0.38 to 0.61)	0.64

Pre- and post-exercise heart rate (HR) refers to the heart rate measured immediately before and after the incremental shuttle walk test. Mean between-group differences (spironolactone minus control level) are given with 95% confidence interval and the significance level. Changes are adjusted for the baseline value, sex, age, and body mass index. Significance of the within-group changes: *P ≤ 0.05

### BP during ISWT

In unadjusted analyses, the correlations between the exercise-induced BP increases and the number of completed shuttles were similar in both groups (Supplementary Table 2). Furthermore, the correlations between baseline BMI and the resting systolic and diastolic BP and the exercise-induced changes in systolic/diastolic BP at months 1 and 9 ranged from −0.16 to 0.15 without any between-group difference (*P* ≥ 0.052).

**Fig. 3:**
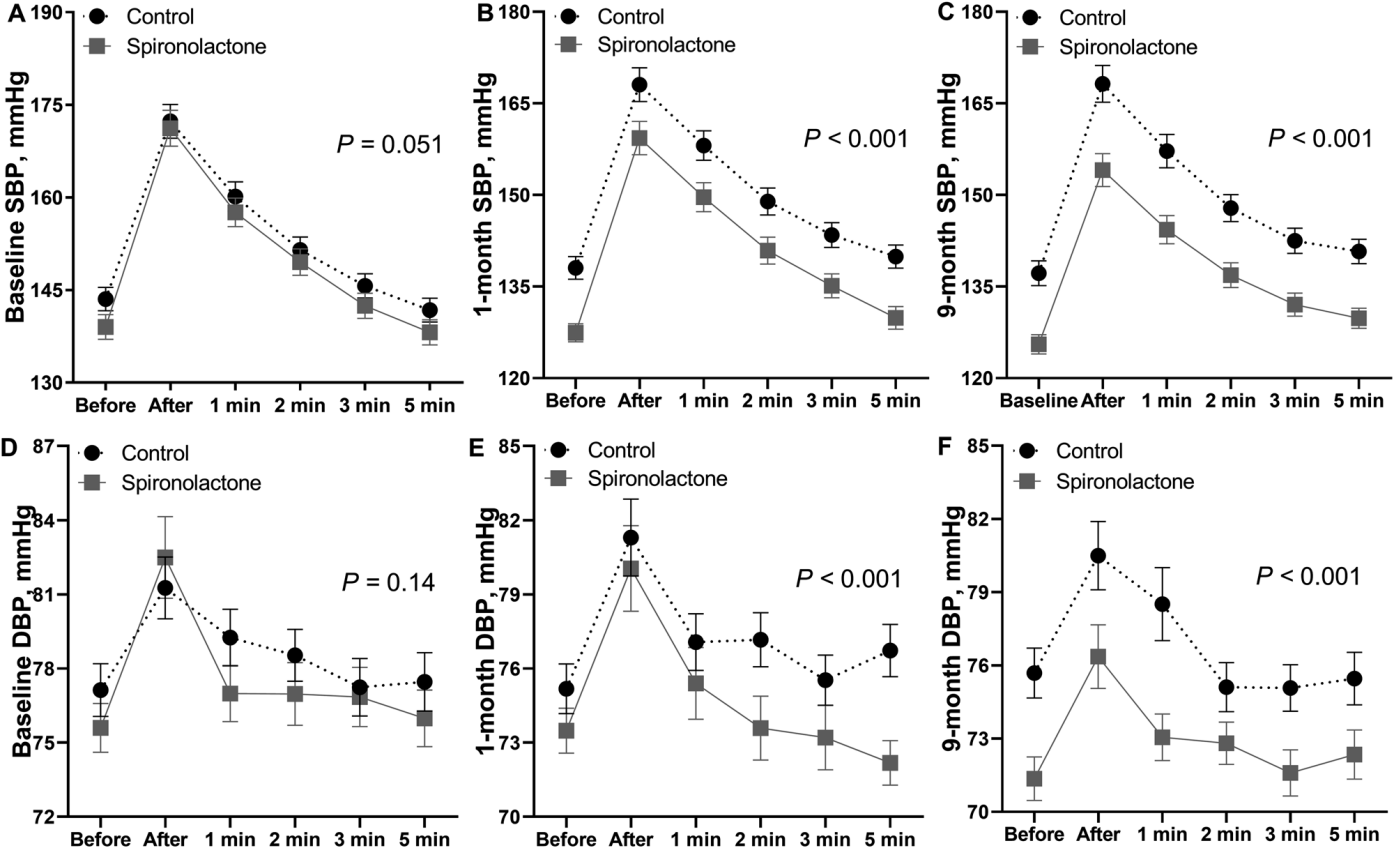
Systolic BP (**A**–**C**) and diastolic BP (**D**–**F**) pre-exercise, immediately post-exercise, and 1, 2, 3 and 5 min after exercise. Data points are unadjusted means ± SE. *P*-values for the between-group difference were computed by repeated measures ANOVA

As shown in Fig. 3 for systolic and diastolic BP, repeated measures ANOVA did not reveal a significant between-group difference at the baseline visit in the pre-exercise BP and the post-exercise BP up to 5 min after completion of the ISWT (*P* ≥ 0.051). However, at months 1 and 9, BP throughout the ISWT was significantly lower in the spironolactone group compared to control (*P* < 0.001; Fig. 3). Adjustment for sex, age, and body mass index produced confirmatory results.

### Analysis of accessory variables

Table 3 in the Supplementary Information summarizes the effects of spironolactone versus control on circulating biomarkers, serum electrolytes, echocardiographic functional measurements, and QoL. There were no differences between control and spironolactone in the QoL changes at 1 and 9 months (Supplementary Table 3). To assess the effects of spironolactone versus control in more detail, correlations were computed between the changes from baseline to the 9-month visit in the pre- and post-exercise systolic BP and in the accessory variables (Supplementary Table 4). The between-group differences in these correlations were not significant with the exception for the change in serum potassium in relation to the change in the post-exercise systolic BP at 9 months (*r* = 0.068 versus *r* = −0.254; *P* = 0.015). The side-effects reported by patients were all mild not necessitating withdrawal of patients from the trial. In the active-treatment group, three men experienced loss of libido, gynecomastia, or tenderness of the breast; in the control group no patient reported such complaints.

## Discussion

To the best of our knowledge, HOMAGE is the first randomized clinical trial allowing to study the effects of usual therapy compared to spironolactone on top of usual therapy on the BP response to ISWT and exercise capacity as captured by the number completed shuttles. Systolic BP measured immediately post-exercise at 1 and 9 months and the diastolic BP at 9 months significantly decreased in the intervention group, irrespective of adjustment (Fig. 2 and Table 2). At months 1 and 9, systolic and diastolic BP throughout the ISWT were lower on spironolactone than in control patients (Fig. 3). The BP-lowering effects of spironolactone on the ISWT-induced BP rise were broadly consistent in analyses dichotomized by sex or the medians of age, LVEF or eGFR (Supplementary Figs. 2–5). Spironolactone did not affect the increase in systolic BP in response to ISWT. In unadjusted analyses, the number of completed shuttles was higher on spironolactone. Adjusted analyses showed a similar trend, albeit that formal significance was lost (Table 2). Spironolactone treatment did not affect QoL. In exploratory analyses, spironolactone did not affect the correlations between the exercise-induced BP changes and the number of completed shuttles (Supplementary Table 2) or the correlations between the changes in the pre- and post-exercise BP at 9 months and the corresponding changes in the accessory measurements (Supplementary Table 4).

In the double-blind placebo-controlled RALES trial of spironolactone (25 mg/day) [15], based on the NYHA classification, three categories were used to assess changes in the symptoms of HF: improvement, no change, and worsening or death. In the placebo group, the condition of 33% of the patients improved, but it did not change in 18%, and it worsened in 48%; in the spironolactone group, the condition improved in 41%, did not change in 21% and worsened in 38%. The between-group difference was significant (*P* < 0.001) [15]. In three double-blind trials with a single-center [18] or multicenter [16, 17] design, 80 [18], 150 [17], or 422 [16] HF patients with preserved LVEF (HFpEF) were randomized to placebo or spironolactone (25 mg/day) and followed up for 6 [17], 9 [18] or 12 [16] months. The number of analyzed patients amounted to 71 [18], 131 [17], and 422 [16], respectively. In the single-center trial (80% women; mean age 71 years) [18], the baseline-adjusted peak VO_2_, was 13.9 mL/min on placebo and 13.5 mL/min on spironolactone, resulting in a between-group difference −0.4 mL/kg/min (95% CI, −1.1 to 0.4 mL/kg/min; *P* = 0.38). There was no improvement in the echocardiographic measurements [18]. In the Polish trial (84% women; mean age: 67 years) [17], spironolactone compared to placebo increased peak VO_2_ (2.9 versus 0.3 mL/min/kg) in relation to a reduction in the exercise-induced E/e’ ratio (−3.0 versus +0.5). In ALDO-DHF (52% women; mean age, 67 years) [16] spironolactone improved LV diastolic function but did not affect VO_2_ or QoL. The adjusted mean between-group difference amounted to −1.5 (95% CI, −2.0 to −0.9; *P* < 0.001) for the E/e’ ratio and −0.1 mL/min/kg (95%CI: −0.6 to −0.8 mL/min/kg; *P* = 0.81) for VO_2_.

Among the aforementioned trials, QoL was either not reported [17] or did not change [16, 18]. However, in the EPHESUS study (39% women; mean age, 64 years), 6632 patients with reduced LVEF (HFrEF) following myocardial infarction were randomized to placebo or eplerenone (25–50 mg/day) and followed up for 16 months on average [19]. Using the Swiss insurance system as reference, eplerenone was cost-effective in reducing mortality and increasing QoL [29]. Compared to eplerenone, the affinity of spironolactone for androgen, glucocorticoid, and progesterone receptors is 10^2^–10^3^ higher [30], potentially explaining why eplerenone increased QoL in EPHESUS [19], but not in the current study or other trials of spironolactone [16, 18]. In a trial set up to investigated whether treatment with spironolactone might be meaningful in the prevention of HF, community-dwelling subjects aged ≥65 years old, with at least one non-ischemic HF risk factor (hypertension, type-2 diabetes, or obesity) were randomized to echocardiography-guided therapy or usual care. LV dysfunction resolved more frequently with spironolactone than in untreated patients [31]. However, the study was underpowered to determine whether screening-guided spironolactone therapy reduced incident HF because spironolactone was frequently discontinued due to the too strict renal function criteria [31].

The current analyses highlighted the signature of mineralocorticoid antagonism (MRA) by spironolactone on serum electrolytes, eGFR, and the circulating fibrosis biomarkers. Neither the increase in serum potassium nor the decrease in eGFR limited the beneficial effects of spironolactone on the pre- and post-exercise BP, the decrease in serum NT-proBNP or the shift in PICP and the PICP/CITP ratio indicative of an antifibrotic effect (Supplementary Fig. 1) [32]. BP lowering is probably a major contributor to the beneficial effects of MRA in HF patients [14–19] or the HOMAGE patients at risk of HF because of coronary heart disease. In HFpEF patients, greater time within the target systolic BP range is associated with a decreased risk of adverse cardiovascular outcomes and mortality, especially among younger patients [33]. In HFpEF patients, abnormal sodium burden, assessed from the serum sodium concentration [34], is an independent predictor of long-term all-cause mortality and HF hospitalization. However, as shown by the PATHWAY-2 trial [35] and in keeping with the lowering of serum sodium, spironolactone is effective in mitigating the adverse effects of the increased sodium load. In this 3-month double-bind trial, 25% of 126 patients enrolled in an endocrinal substudy (*n* = 31) had an inappropriately elevated aldosterone as assessed by ratio of plasma aldosterone to plasma renin and a plasma aldosterone exceeding the mean value in all 126 patients (250 pmol/L [9 ng/dL]). In these 31 patients, spironolactone 25 mg/d, compared with placebo, reduced systolic BP by 18.3 mm Hg (95%CI, 16.2–20.5 mm Hg) and the thoracic fluid content by 6.8% (95% CI, 4.0–8.8%) [35]. In TOPCAT-Americas [36] compared to placebo, spironolactone increased worsening of renal function (doubling of serum creatinine), but this feature did not preclude substantial beneficial effects in terms of lowering the risk of cardiovascular and all-cause mortality. In the current study, the BP lowering effects of spironolactone were similar if dichotomized by median eGFR (Supplementary Fig. 5).

### Study limitations

This report on a subset of patients from the HOMAGE trial has several limitations. First, we excluded patients, who did not complete three ISWTs, i.e., at months 0, 1, and 9. However, not including the same patients at each time point would have precluded a direct comparison of the sort- and long-term results. Second, the between-group differences in the number of completed shuttles (Table 2) or the changes in QoL (Supplementary Table 3) did not reach significance, probably because of a lack of statistical power, given that only 227 of the 527 [23] (43.0%) HOMAGE patients were analyzed with a follow-up not longer than 9 months. Spironolactone-specific complaints include loss of libido, gynecomastia, or tenderness of the breast [30], but occurred only in three men and did not explain the absence of an effect of active treatment on QoL. The number of shuttles completed and the self-assessed QoL were accessory variables. The trial’s primary endpoint was the interaction between PIINP (Supplementary Fig. 1) and galectin-3 [23]. Although the analyses of exercise capacity and QoL were prespecified according to the HOMAGE protocol [22], the power to detect a significant difference, assuming an effect size of 20% with a 2-sided α-level of 0.05, was low, respectively, 0.05 for the number of completed shuttles and 0.20 for QoL. These HOMAGE result should therefore be considered as exploratory because they do not exclude a type I error, albeit that these outcomes are in line with other trials of spironolactone [16, 18]. Third, women were underrepresented in our study, to some extent limiting its generalizability. Fourth, BP was measured in the clinics during the trial visits, which could have biased the BP values compared to out-of-office BP levels. However, due to consistency of the BP effects in subgroups and the replication of the BP results in other HF cohorts [37], these findings might be regarded as robust. Fifth, the clinical centers were free to organize the ISWT according to the local working routines irrespective of the time interval between the intake of medications, including spironolactone in the active-treatment group and the test. However, spironolactone undergoes rapid extensive metabolism to three active metabolites with prolonged half-lives (13.8–16.5 h) [30], so that this feature of conducting the trial is unlikely to have biased the results. Finally, given the relatively small sample size and the short follow-up, we could not relate the ISWT results to outcome. However, there are multiple long-term studies in patients [10, 38, 39] showing that exercise capacity as assessed by the ISWT predicts mortality [38, 39] and cardiovascular [39] or cardiac [10] endpoints.

## Conclusions

In patients at increased risk of developing HF, MRA by spironolactone reduced the pre- and post-exercise BP but did not result in significant increases in exercise capacity or QoL. Guidelines for the management of symptomatic HF (stages III-IV) [40, 41] unanimously recommend the use of MRAs giving the overwhelming evidence from randomized clinical trials [14–21]. Given the current results, use of spironolactone 25–50 mg/day may also be considered in the management of patients at increased risk of developing HF, because of coronary heart disease; its use is not limited by hyperkalemia or an excessive reduction in eGFR.

## Supplementary Information

Data Supplement

List of HOMAGE Investigators
