# Supplementary material for: Effects of spironolactone on exercise blood pressure in patients at increased risk of developing heart failure: report from the HOMAGE trial
Source: Hypertens Res. 2024 Sep 6;47(11):3225–36. doi: 10.1038/s41440-024-01843-z (PMC11534698; doi:10.1038/s41440-024-01843-z)
Supplement: Supplementary file 1 — Data Supplement [file 41440_2024_1843_MOESM1_ESM.doc]

***Hypertens Res***

**Data Supplement**

**Effects of spironolactone on exercise blood pressure in patients at increased risk of
developing heart failure: report from the HOMAGE trial**

Wei FF, Pellicori P, Ferreira JP, González A; Mariottoni B,
An DW, Verdonschot JAJ, Liu C, Ahmed FZ, Petutschnigg J, Rossignol P,
Heymans H, Cuthbert J, Girerd G, Clark AL, Li Y, Nawrot TS, Díez J,
Zannad F, Cleland JGF, Staessen JA, on behalf of the HOMAGE investigators

**Table of contents**

**HOMAGE investigators** p2

***Protocol of the incremental shuttle walk tes*t** p3

***Supplementary Table 1*** Baseline characteristics of patients analyzed and not analyzed p8

***Supplementary Table 2*** Correlations between the number of completed shuttles and
exercise-induced BP changes by treatment group p9

***Supplementary Table 3*** Biochemical and clinical biomarkers at randomization and at
months 1 and 9 by treatment group p10

***Supplementary Table 4*** Correlations between changes in biomarkers and in SBP
at month 9 by treatment group p12

***Supplementary Figure 1*** Circulating biomarkers of collagen turnover p13

***Supplementary Figure 2*** Changes in the pre-exercise (A,B) and post-exercise (C,D) SBP
from baseline to months 1 and 9 by randomization group and sex p14

***Supplementary Figure 3*** Changes in the pre- and post-exercise SBP from baseline
to months 1 and 9 by randomization group and median age p15

***Supplementary Figure 4*** Changes in the pre- and post-exercise SBP from baseline
to months 1 and 9 by randomization group and median LVEF p16

***Supplementary Figure 5*** Changes in the pre- and post-exercise SBP from baseline
to months 1 and 9 by randomization group and median eGFR p17

**HOMAGE investigators**

*Robertson Centre for Biostatistics, Institute of Health and Wellbeing, University of Glasgow, Glasgow, Scotland, UK* — John GF Cleland, MD; Pierpaolo Pellicori, MD; Javed Khan, MD.

*Université de Lorraine, Inserm, Centre d’Investigation Clinique Plurithématique, CHRU de Nancy, Nancy, France* — João P Ferreira, MD; Franco Cosmi, MD; Anne Pizard, PhD; Nicolas Girerd, MD; Patrick Rossignol, MD; Erwan Bozec, MD; María U Moreno, MD; Faiez Zannad, MD.

*Department of Cardiology, Cortona Hospital, Arezzo, Italy* — Beatrice Mariottoni, MD.

*Department of Cardiology, University of Hull, Castle Hill Hospital, Cottingham, East Riding of Yorkshire, UK* — Joe Cuthbert, MD; Andrew L Clark, MD.

*Department of Cardiology, Maastricht University Medical Center, Maastricht, the Netherlands* — Job AJ Verdonschot PhD; Hans P Brunner La Rocca, MD; Mark Hazebroek, PhD; Stephane Heymans, MD.

*Department of Internal Medicine and Cardiology, Campus Virchow Klinikum, Charité‚ University Medicine Berlin, Berlin Institute of Health (BIH), and German Centre for Cardiovascular research (DZHK), Partner Site Berlin, Germany* — Johannes Petutschnigg, MD; Frank Edelmann, MD; Burkert Pieske, MD.

*Division of Cardiovascular Sciences, School of Medical Sciences, Faculty of Biology, Medicine and Health, Manchester Academic Health Science Centre, University of Manchester, Manchester, UK* — Fozia Z Ahmed, MD; Mamas A Mamas, MD.

*Centre for Prognosis Research, Institute for Primary Care and Health Sciences, Keele University, Newcastle, UK* — Mamas A Mamas, MD.

*German Heart Center Berlin, Berlin, Germany* — Burkert Pieske, MD.

*St. Vincent’s University Healthcare Group, and School of Medicine, University College Dublin, Dublin, Ireland* — Ken McDonald, MD.

*Equipe obésité et insuffisance cardiaque, Université Paul Sabatier, Inserm I2MC, Toulouse, France* — Philippe Rouet, MD.

*Studies Coordinating Centre, Research Unit Hypertension and Cardiovascular Epidemiology, Department of Cardiovascular Sciences, University of Leuven, Leuven, Belgium* — L Thijs, MSc.

*Non-Profit Research Association Alliance for the Promotion of Preventive Medicine, Mechelen, Belgium* — Jan A Staessen, MD; Kei Asayama, MD; Tine W Hansen, MD; Gladys E Maestre, MD.

*Program of Cardiovascular Diseases, CIMA. Universidad de Navarra and IdiSNA, Pamplona, Spain CIBERCV, Carlos III Institute of Health, Madrid, Spain* — Arantxa González, PhD; Suzanna Ravassa, PhD; Begoñia López, PhD; Javier Díez, MD.

*Departments of Nephrology and Cardiology, Clínica Universidad de Navarra, Pamplona, Spain* — Javier Díez, MD.

*Department of Cardiovascular Medicine,* *Istituto di Ricerche Farmacologiche Mario Negri – IRCCS, Milan, Italy* —Roberto Latini, MD.

*Fondation Force, Research and Consulting Department, EDDH, Centre de Médecine Préventive, Vandoeuvre les Nancy, France* — Stephanie Grojean, PhD.

*Department of Medical Statistics, London School of Hygiene and Tropical Medicine, London, UK* — Tim Collier, PhD.

**Protocol of the ISWT**

**Introduction**

Exercise requires more oxygen to be delivered to skeletal muscles. This is achieved by an increase in heart rate and stroke volume, resulting in greater cardiac output, a redistribution of blood flow from other vascular beds, an increase in oxygen uptake achieved by faster respiratory rate, higher pulmonary blood flow and ventilation. The incremental shuttle walk-test (ISWT) is designed to provoke symptoms and assess maximum exercise capacity. The 6-minute walking test (6MWT) is often a longer test better designed to assess sub-maximal exercise capacity. The ISWT is a better method for grading the cardiorespiratory performance and to engage in physical effort. The symptoms and signs of heart failure are often only manifest during exercise. Many patients with cardiac dysfunction avoid provoking symptoms by reducing or avoiding physical exertion. Some form of exercise testing should be a routine part of the assessment of any person with, or at risk of developing, heart failure.

The ISWT is a standardized externally-paced, progressive, incremental field walking test for patients comprising up to 12 levels and 102 shuttles. Patients walk around a 10‑metre shuttle course marked by cones in time with a series of bleeps played from a CD. The ISWT is a valid has been shown to be a valid and reproducible measure of exercise capacity in patients with chronic obstructive pulmonary disease. It produces a higher peak heart-rate and Borg dyspnea score as well as a more graded cardiorespiratory response to exercise compared to the 6MWT. It is used as an outcome measure of exercise capacity in pulmonary rehabilitation but has also been used to assess patients with heart failure.^1,2^ Changes of 50-70 meters are considered clinically significant.^3^ There is a learning effect. When using the ISWT as a trial endpoint, the patient should have at least one test prior to the baseline test.

**Required equipment**

The required equipment consists of two cones, two chairs, stopwatch, CD player with ISWT CD, typed ISWT instructions, typed instructions, and an assessment sheet.

**Contraindications and precautions**

Unstable angina or myocardial infarction during the previous month are absolute contra-indications for the ISWT, while a resting heart rate of more than 120 beats per minute and a systolic blood pressure of more than 180 mm Hg are relative contraindications. Patients with any of these findings should be referred to the physician ordering or supervising the test for individual clinical assessment and a decision about the conduct of the test. The results from a resting electrocardiogram done during the previous 6 months should also be reviewed before testing. Stable exertional angina is not an absolute contraindication for an ISWT, but patients with these symptoms should perform the test, after using their anti-angina medication and rescue nitrate medication should be readily available.

Testing should be performed in a location where a rapid, appropriate response to an emergency is possible. The appropriate location of the crash trolley should be determined by the assessor undertaking the test. At least one other person should be within earshot should an emergency arise. Oxygen must be available. If applicable, the patient’s sublingual nitroglycerine and or salbutamol, must also be accessible. A telephone or other means of calling help should be available. The person doing the test should have training in cardiopulmonary resuscitation and at least one other trained individual should be in the vicinity. The assessor should be completely familiar with the ISWT procedures and have had one or more practice runs before administering the test to a patient.

**Before the test**

The observer should clearly explain and demonstrate all procedures prior to testing and ensure the patient understands the instructions. If a patient is uncomfortable performing a test or if the observer feels that it is not safe for an individual to continue, the test should not be performed. The reason for not performing the test should be documented.

- If this is the patient’s initial assessment, document the past-medical history.
- Identify and record if the patient will do the test using a walking aid.
- Measure blood pressure, heart-rate, resting oxygen saturations and Borg dyspnea level.
- If at the initial assessment the patients resting oxygen saturations is less than 92% consider assessment for respiratory disease.
- Set-up the course: two cones placed 9 meters apart on flat, straight flooring with a chair 1 meter behind each cone (f**igure**).
- Show the patient the course and play the standardized instructions on the CD player or read the typed standardized instructions to the patient.
- Ask the patient if she/he understands the instructions and answer any questions asked.
- Before the test starts remind the patient that “*this is a maximal test, by the end of the test you should walk or run as fast as you can*”.

**During the test**

- Walk with the patient for the first level (3 shuttles) to ensure correct pacing.
- The standardized instructions should be administered as appropriate and the patient should not be encouraged. It is aal right to advise the patient to slow her/his walking speed to ensure a continuous walk.
- At each new level (triple bleep) instruct the patient “*to increase your speed now*”.
- Record each completed shuttle
- If the patient is less than 0.5 meter from the cone do not say anything.
- If the patient is greater than 0.5 meter from the cone advise the patient that “*You’re not going fast enough, try to make up the speed now*”.

**The test can be terminated by the patient**

- The patient feels unable to keep up.
- Limiting symptoms may be breathlessness, fatigue, claudication, chest pain, dizziness or other reasons that concern the test supervisor.
- The person supervising the test is concerned about patient safety.
- The patient is greater than 2 meters from the cone two shuttles in a row, i.e., the observer has given the patient a chance to increase her/his speed, but she/he is still not within 2.0 meters of the cone.

**After the test**

- Sit the patient on a chair − if patient is able to walk to one of the chairs behind the cones or if the patient is unable to walk bring a chair to the patient.
- Immediately record heart and respiratory rate and Borg dyspnea level.
- Immediately begin timing recovery for five minutes.
- Record completed shuttles only, excluding the last shuttle it was terminated because the patient was more than 2.0 meters from the cone.
- Record why the test was stopped.

**Verbal instructions for the ISWT**

- The object of the progressive shuttle walking test is to walk for as long as possible back and forth along the 10-metre course keeping to the speed indicated by the beeps on the CD. You will hear these beeps at regular intervals. You should walk at a steady pace aiming to turn around the cone at one end of the course when you hear the first beep and at the other end when you hear the next.
- At first your walking speed will be very slow but you will need to speed up at the end of each minute. Your aim should be to follow the set rhythm for as long as you can. Each single beep signals the end of a shuttle and each triple beep signals an increase in walking speed. You should stop walking only when you become too breathless to maintain the required speed or can no longer keep up with the set pace.
- The test is maximal and progressive, in other words, it is easier at the start and harder at the end. Your walking speed for the first minute is very slow and you have 20 seconds to complete each 10-metre shuttle, so do not go too fast.
- Level one starts with a triple beep after the 4 second count down.
- Note to the assessor: before you start the ISWT remind patient that *“this is a maximal test, by the end of the test you should walk or run as fast as you can”.*

**References**

1. Lewis ME, Newall C, Townend JN, Hill SL, Bonser RS. Incremental shuttle walk test in the assessment of patients for heart transplantation. *Heart.* 2001;86:183-187.

2. Malkin CJ, Pugh PJ, West JN, van Beek EJR, Jones TH, Channer KS. Testosterone therapy in men with moderate severity heart failure : a double-blind randomized placebo-controlled trial. *Eur Heart J.* 2006;27:57-64.

3. Singh SJ, Jones PW, Evans R, Morgan MDL. Minimum clinically improvement for the incremental shuttle walking test. *Thorax.* 2008;63:775-777.

Supplementary Table 1

**. Baseline Characteristics of Included and Excluded Patients.**

| **Characteristic** |  | **Included** | **Not included** | ***P* Value** |
| --- | --- | --- | --- | --- |
| **Number with characteristics** |  | 227 | 300 |  |
| Women |  | 49 (21.6) | 86 (28.7) | 0.065 |
| Caucasian |  | 221 (97.8) | 298 (99.3) | 0.35 |
| Current smoking |  | 13 (5.73) | 31 (10.3) | 0.040 |
| Hypertension |  | 171 (75.3) | 242 (80.7) | 0.14 |
| Treated hypertension |  | 207 (91.2) | 272 (90.7) | 0.84 |
| Diabetes |  | 87 (38.3) | 130 (43.3) | 0.25 |
| Treated diabetes |  | 84 (37.0) | 108 (37.4) | 0.93 |
| Ischaemic heart disease |  | 180 (79.3) | 199 (66.3) | 0.001 |
| History of myocardial infarction |  | 93 (51.7) | 121 (60.5) | 0.083 |
| **Clinical characteristics** |  |  |  |  |
| Age (years) |  | 72.9±6.07 | 75.2±6.79 | <0.001 |
| BMI (kg/m^2^) |  | 29.2±5.20 | 28.7±4.91 | 0.26 |
| Waist-to-hip ratio |  | 0.98±0.07 | 0.98±0.06 | 0.73 |
| **Biochemistry** |  |  |  |  |
| Serum sodium (mmol/L) |  | 138.8±2.72 | 139.7±2.74 | <0.001 |
| Serum potassium (mmol/L) |  | 4.33±0.36 | 4.31±0.36 | 0.53 |
| eGFR (mL/min/1.73 m^2^) |  | 73.1±16.3 | 68.8±15.5 | 0.002 |

Values are number of patients (%) or mean± SD. The glomerular filtration rate is derived from serum creatinine by the Chronic Kidney Disease Epidemiology Collaboration equation.

Supplementary Table 2

**. Correlations Between Completed Shuttle Numbers and Exercise‑Induced BP Change by Treatment Group**

| Study phase | **Control** | **Spironolactone** | ***P* Value** |
| --- | --- | --- | --- |
| At baseline |  |  |  |
| ∆SBP | 0.49‡ | 0.49‡ | >0.99 |
| ∆DBP | 0.19* | 0.25† | 0.64 |
| At 1-month |  |  |  |
| ∆SBP | 0.45‡ | 0.47‡ | 0.85 |
| ∆DBP | 0.24† | 0.24* | >0.99 |
| At 9-month |  |  |  |
| ∆SBP | 0.52‡ | 0.40‡ | 0.26 |
| ∆DBP | 0.16 | 0.26 | 0.44 |

*P*-values indicate the significance of the difference in the correlation coefficients obtained by Fisher z-transform. Significance of the within-group correlations: * *P*≤0.05; † *P*≤0.01; ‡ *P*≤0.001.

Supplementary Table 3

**. Biochemical and Clinical Biomarkers at Randomization and at 1 and 9 Months by Treatment Group (Starts)**

| **Variable** | **Control  (n=113)** | **Spironolactone  (n=114)** | **Difference  (95% CI)** | ***P* Value** |
| --- | --- | --- | --- | --- |
| Circulating biomarkers |  |  |  |  |
| PICP, μg/L |  |  |  |  |
| Baseline | 77.5 (66.7 to 90.7) | 76.8 (62.3 to 91.8) | -0.90 (-7.57 to 6.27) | 0.80 |
| Change at 9 months, % | 1.06 (-5.71 to 3.85) | -9.31 (-13.6 to -4.72)‡ | -10.3 (-15.6 to -4.57) | <0.001 |
| PICP/CITP ratio |  |  |  |  |
| Baseline | 23.1±9.31 | 24.2±10.4 | 1.16 (-1.43, 3.75) | 0.38 |
| Change at 9 months | -1.23±0.76 | -3.28±0.76‡ | -2.05 (-3.97, -0.14) | 0.036 |
| NT-proBNP, ng/L |  |  |  |  |
| Baseline | 190 (119 to 288) | 200 (120 to 331) | 4.88 (-12.7 to 25.9) | 0.61 |
| Change at 9 months, % | 6.24 (-3.97 to 17.5) | -9.22 (-18.0 to 5.77) | -14.5 (-24.8 to -2.75) | 0.017 |
| Serum sodium, mmol/L |  |  |  |  |
| Baseline | 138.9±2.60 | 138.6±2.84 | -0.31 (-1.02, 0.41) | 0.40 |
| Change at 9 months | 0.17±0.21 | -1.28±0.21‡ | -1.45 (-1.98, -0.91) | <0.001 |
| Serum potassium, mmol/L |  |  |  |  |
| Baseline | 4.35±0.35 | 4.31±0.37 | -0.04 (-0.13, 0.06) | 0.42 |
| Change at 9 months | 0.04±0.04 | 0.22±0.04‡ | 0.18 (0.07, 0.29) | <0.001 |
| Echocardiographic traits |  |  |  |  |
| E/e’ |  |  |  |  |
| Baseline | 9.71±3.68 | 9.63±2.96 | -0.08 (-0.99, 0.83) | 0.86 |
| Change at 9 months | 0.48±0.26 | -0.09±0.26 | -0.57 (-1.22, 0.08) | 0.085 |

Supplementary Table 3

**.**

**Biochemical and Clinical Biomarkers at Randomization and at 1 and 9 Months by Treatment Group (Continued)**

| **Variable** | **Control  (n=113)** | **Spironolactone  (n=114)** | **Difference  (95% CI)** | ***P* Value** |
| --- | --- | --- | --- | --- |
| LAVI, mL/m2 |  |  |  |  |
| Baseline | 30.5±8.09 | 31.2±7.46 | 0.71 (-1.51, 2.93) | 0.53 |
| Change at 9 months | -0.14±0.51 | -1.20±0.51* | -1.06 (-2.48, 0.37) | 0.15 |
| LVMI, g/m2 |  |  |  |  |
| Baseline | 98.3±27.9 | 96.0±22.7 | -2.30 (-9.17, 4.57) | 0.51 |
| Change at 9 months | 0.53±1.10 | -3.98±1.09‡ | -4.51 (-7.57, -1.45) | 0.004 |
| eGFR, mL/min/1.73 m2 |  |  |  |  |
| Baseline | 71.2±15.2 | 75.0±16.8 | 3.83 (-0.37, 8.02) | 0.074 |
| Change at 9 months | -1.33±0.76 | -6.15±0.76‡ | -4.81 (-6.95, -2.68) | <0.001 |
| Self-rated QoL, points |  |  |  |  |
| Baseline | 2 (1,5) | 3 (1,5) | 0.09 (-0.06, 0.24) | 0.22 |
| Change at 9 months | -0.15 (-0.52, 0.22) | -0.48 (-0.86, -0.11)* | -0.33 (-0.81, 0.14) | 0.17 |

Abbreviations: PICP, procollagen type I carboxy-terminal propeptide; NT-proBNP, N-terminal pro-brain natriuretic peptide; E/e’, ratio of the peak velocity of the early diastolic transmitral blood flow to the peak velocity of the early mitral annular movement; LAVI, left atrial volume index, LVMI, left ventricular mass index; eGFR, glomerular filtration rate estimated from serum creatinine; and QoL, quality of life as assessed by the EQ5D visual analogue score (range: 1-9). Mean between-group differences are given with 95% confidence interval and the significance level. Prior to analysis, PICP and NT‑proBNP were logarithmically transformed (basis 10) to approximate the normal distribution. The central tendency and spread are reported as geometric mean (IQR) for PICP and NT‑proBNP, as arithmetic (±SD) means for the PICP/CITP ratio and the echocardiographic data, and as median (IQR) for the self-rated QoL. The between-group differences are calculated by subtracting the mean changes from baseline in the control group from the corresponding changes in the spironolactone group. Within-group and between-group differences for logarithmically transformed variables were expressed as percentage. Changes are adjusted for the baseline value, sex, age and body mass index. LAVI and LVMI are only adjusted for baseline and age, because these measurements are standardized to body surface area. Significance of the within-group changes: * *P*≤0.05; ‡ *P*≤0.001.

Supplementary Table 4

**. Correlations Between Changes in Biomarkers and in SBP at 9 Months by Treatment Group**

| **Variable** | **Change in resting SBP at 9-month** | | | **Change in post-exercise SBP at 9-month** | | |
| --- | --- | --- | --- | --- | --- | --- |
|  | **Control** | **Spironolactone** | ***P* Value** | **Control** | **Spironolactone** | ***P* Value** |
| Circulating biomarkers |  |  |  |  |  |  |
| PICP, % | 0.159 | 0.016 | 0.29 | 0.033 | -0.002 | 0.99 |
| CITP, % | -0.100 | -0.067 | 0.47 | -0.089 | -0.034 | 0.87 |
| PICP/CITP ratio | 0.167 | 0.011 | 0.25 | 0.103 | 0.016 | 0.50 |
| NT-proBNP, % | 0.250† | -0.020 | 0.081 | 0.089 | -0.096 | 0.43 |
| Serum Na^+^, mmol/L | -0.044 | 0.189* | 0.080 | -0.106 | 0.131 | 0.077 |
| Serum K^+^, mmol/L | -0.073 | -0.263† | 0.14 | 0.068 | -0.254† | 0.015 |
| Echocardiographic traits |  |  |  |  |  |  |
| LVMI, g/m2 | 0.209* | -0.045 | 0.078 | 0.196 | -0.016 | 0.14 |
| E/e’ | 0.127 | -0.021 | 0.31 | -0.057 | -0.066 | 0.95 |
| LAVI, mL/m2 | 0.012 | 0.081 | 0.67 | 0.001 | -0.121 | 0.45 |
| eGFR, mL/min/1.73 m2 | 0.176 | 0.145 | 0.81 | 0.101 | 0.105 | 0.98 |
| Completed shuttles, n | -0.010 | 0.059 | 0.61 | 0.176 | 0.231* | 0.67 |
| Self-rated QoL, points | -0.107 | 0.029 | 0.31 | 0.087 | -0.073 | 0.23 |

For abbreviations, see Supplementary Table 3. Changes were obtained by subtracting baseline from the 9-month value. For PICP, CITP and NT‑proBNP, which were logarithmically transformed (basis 10) to approximate the normal distribution, changes are expressed in percents. *P*-values indicate the significance of the difference in the correlation coefficients obtained by Fisher z-transform. Significance of the within-group correlations: * *P*≤0.05; † *P*≤0.01.


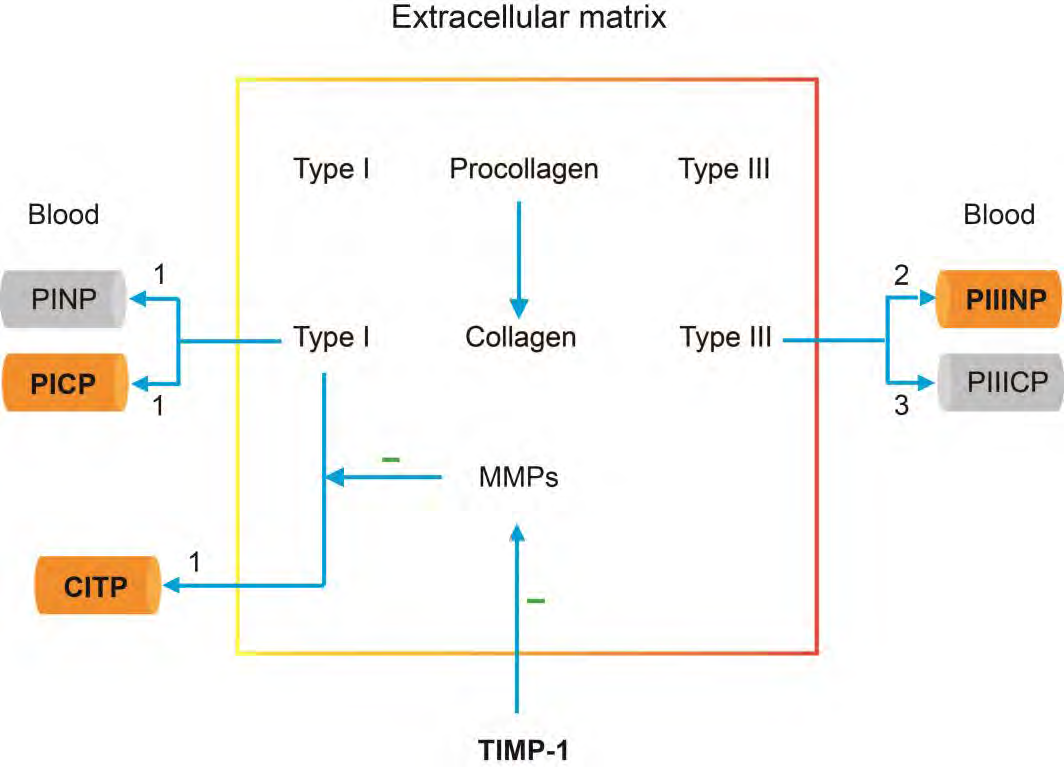


**Supplementary Figure 1**

**.**

**Circulating Biomarkers of Collagen Turnover**

PINP and PICP are released during conversion of procollagen type‑1 to collagen type‑1 and CITP during the degradation of collagen type‑1 by matrix metalloproteinases, which are inhibited by TIMP1. PIIINP and PIIICP are released during conversion of procollagen type‑III to collagen type‑III. Bracketed numbers indicate the stoichiometric ratio. In the present study, serum levels of PICP, CITP, PIIINP, MMP1 and TIMP1 were measured. PICP and CITP are direct indicators of collagen type‑1 synthesis and degradation. PIIINP is an indirect indicator of collagen‑III synthesis, because cleavage at the amino-terminus proceeds at a relatively slow rate and, thus, partially processed procollagen molecules remain bound to the surface of collagen type‑III fibers (*JACC* 2015;65:2449-2456). Abbreviations: PICP, procollagen type‑I carboxy-terminal propeptide; PINP, procollagen type‑I amino-terminal propeptide; CITP, carboxyterminal telopeptide of type‑I collagen; MMPs, matrix metalloproteinases; TIMP1, tissue inhibitor of the matrix metalloproteinase type‑1; PIIICP, procollagen type‑III carboxy-terminal propeptide; and PIIINP, procollagen type‑III amino-terminal propeptide.


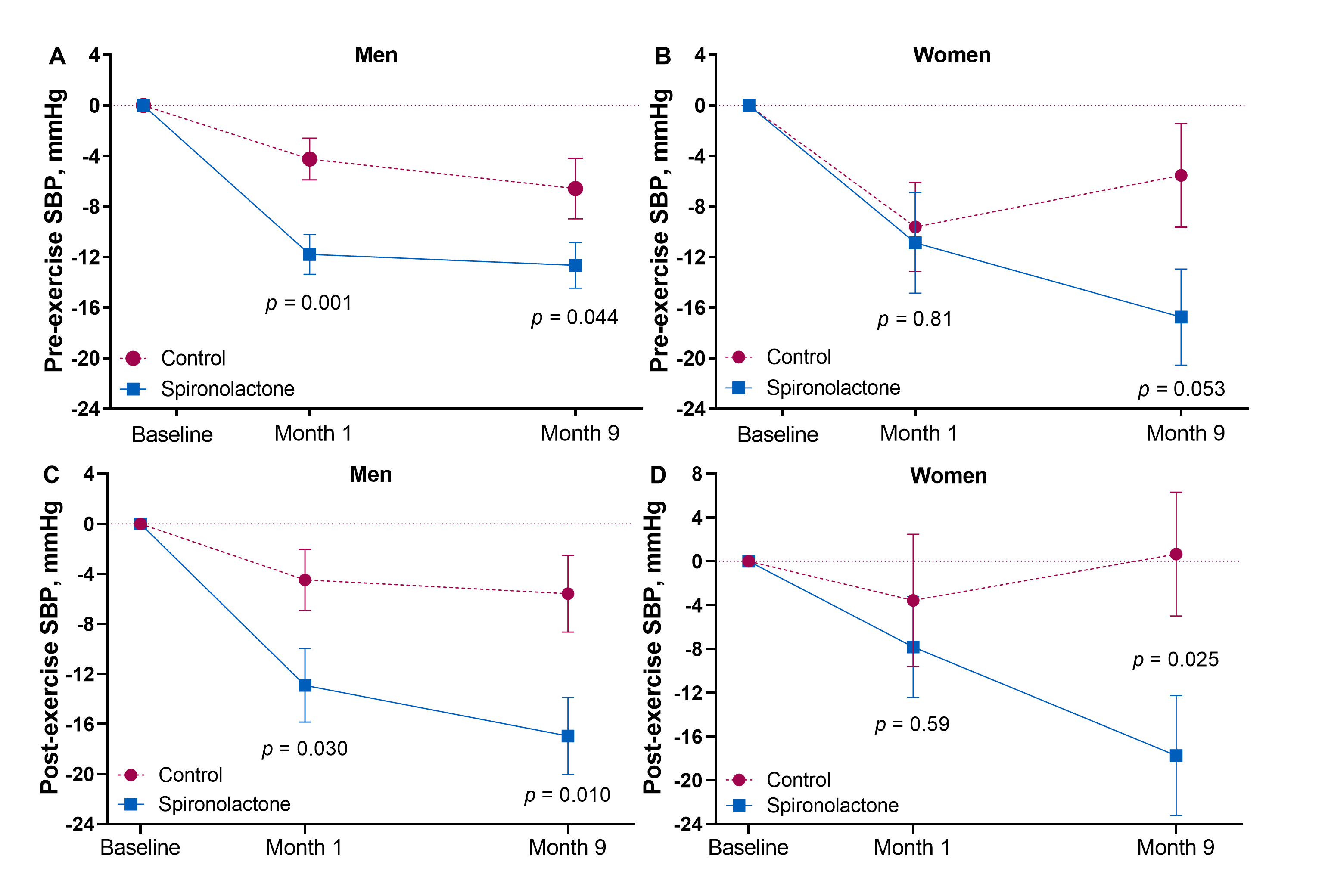


**Supplementary Figure 2.**

**Changes in the Pre-Exercise (A,B) and Post-Exercise (C,D) Systolic BP from Baseline to months 1 and 9 by Randomization Group and Sex**
The sex-by-time interactions were not significant (*P*≥0.62). *P* values in the figure panels refer to the difference between control and spironolactone.


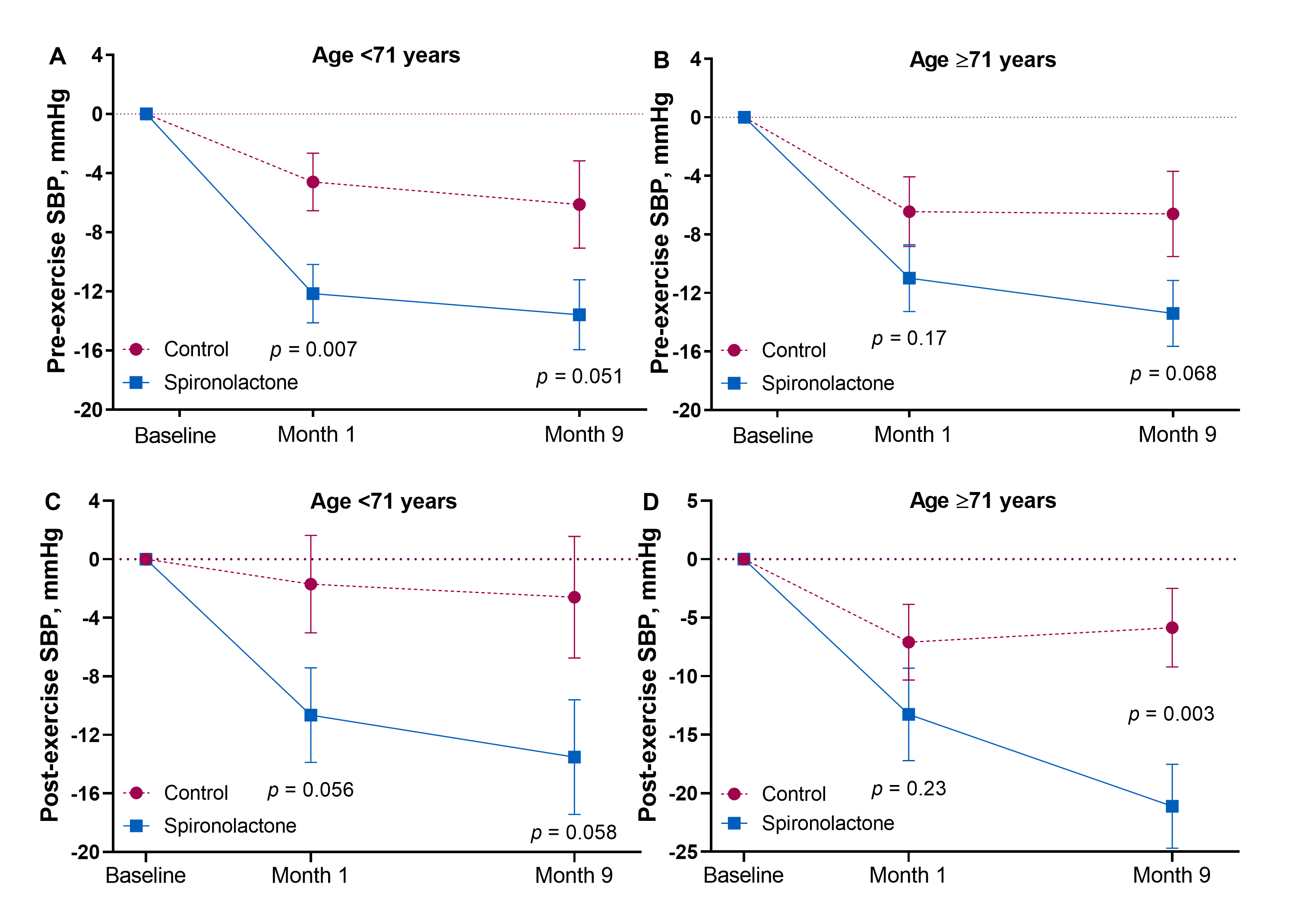


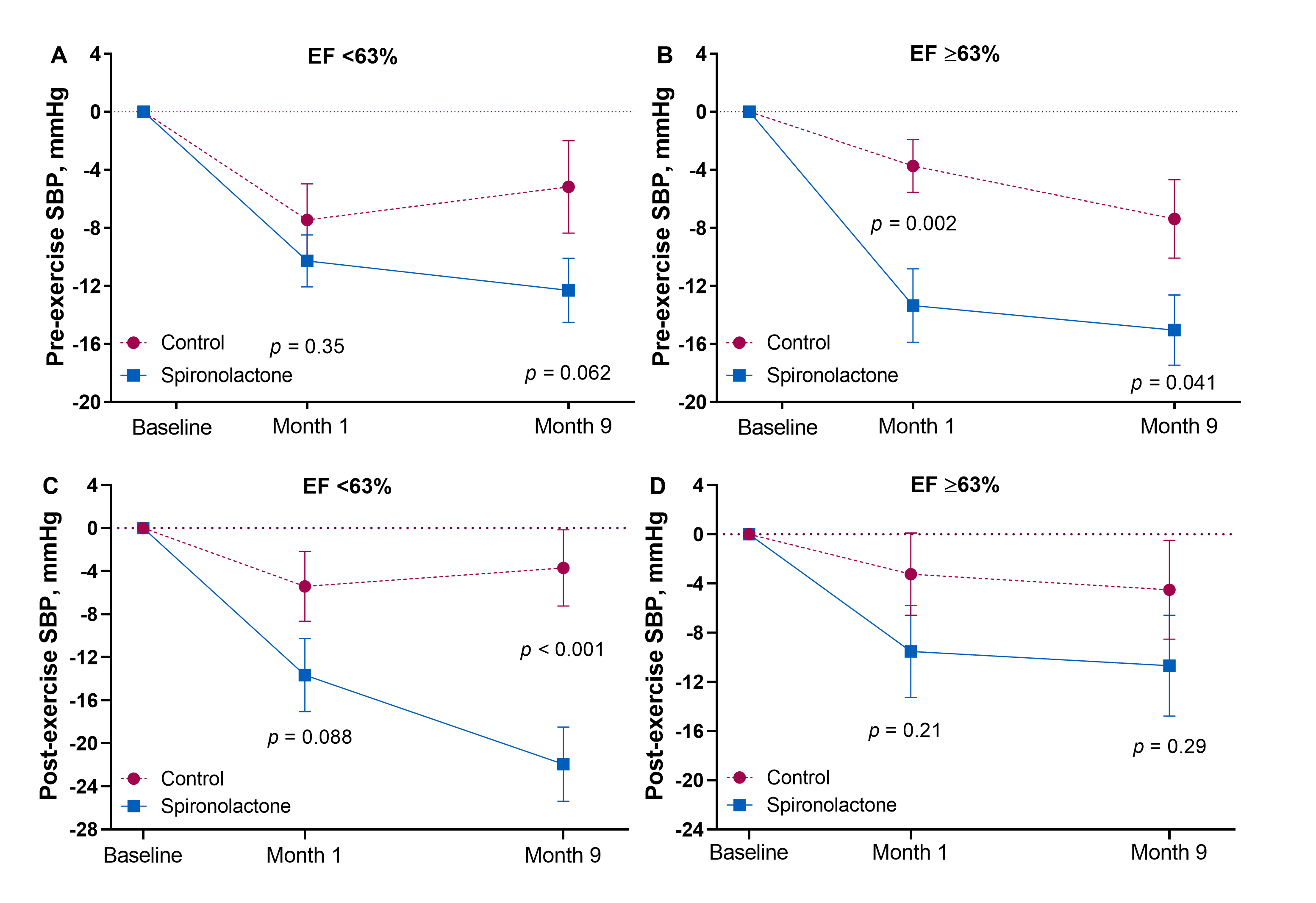


**Supplementary Figure 3.**

**Changes in the Pre-Exercise (A,B) and Post-Exercise (C,D) Systolic BP from Baseline to Months 1 and 9 by Randomization Group and Age**
Changes in the pre-exercise (A,B) and post-exercise (C,D) systolic blood pressure from baseline to month 1 and 9 by randomization group and median age. The time-by-age interaction terms were not significant (*P*≥0.34). *P* values in the figure panels refer to the difference between control and spironolactone.

**Supplementary Figure 4.**

**.**

**Changes in the Pre-Exercise (A,B) and Post-Exercise (C,D) Systolic BP from Baseline to Months 1 and 9 by Randomization Group and Median LVEF**
The time-by-LVEF interaction terms were not significant (*P*≥0.25). *P* values in the figure panels refer to the difference between control and spironolactone.


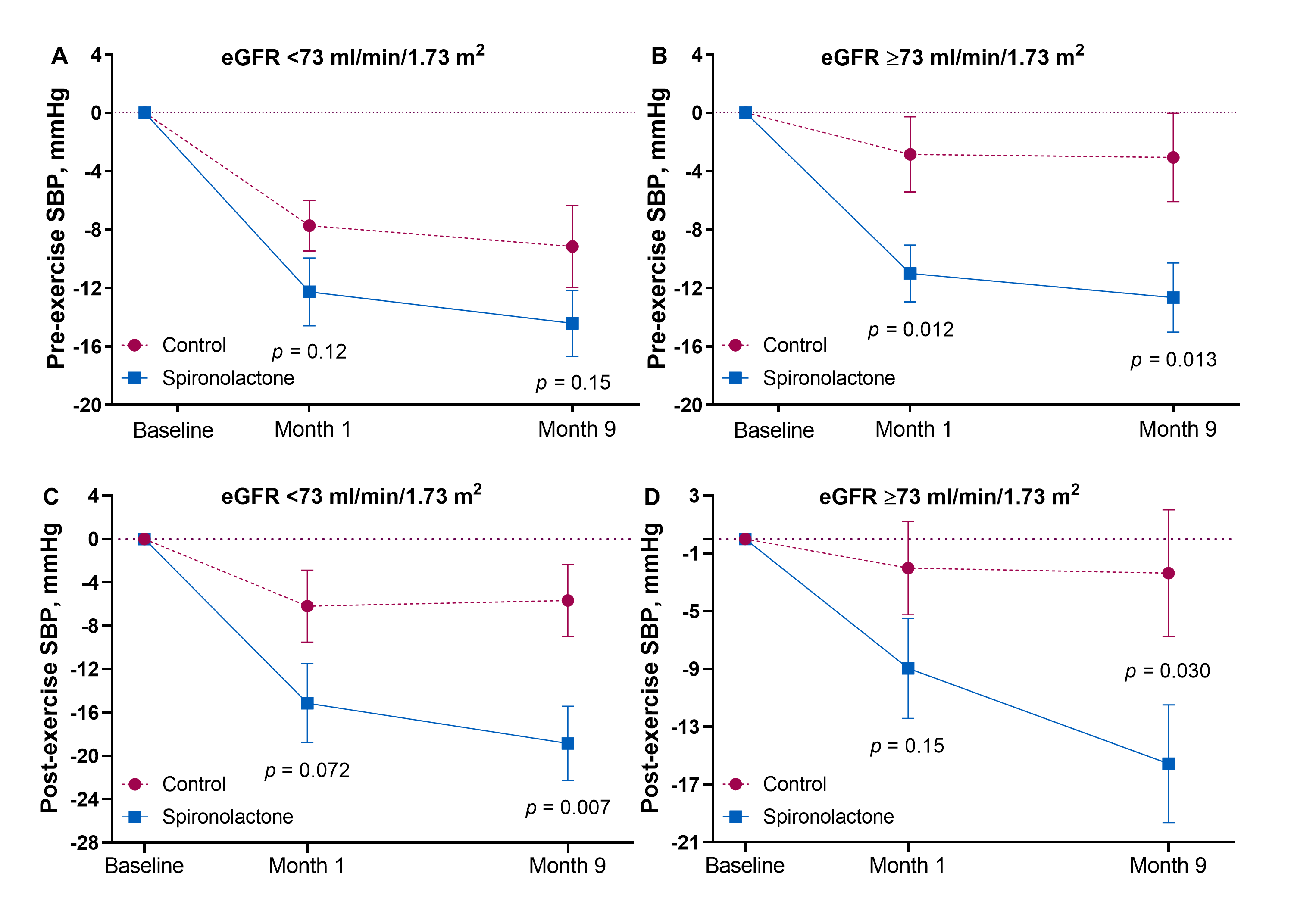


**Supplementary Figure 5.**

**.**

**Changes in the Pre-Exercise (A,B) and Post-Exercise (C,D) Systolic BP from Baseline to Months 1 and 9 by Randomization Group and Median eGFR**The time-by-eGFR interaction terms were not significant (*P*≥0.77). *P* values in the figure panels refer to the difference between control and spironolactone.
