# Supplementary material for: Effects of spironolactone on exercise blood pressure in patients at increased risk of developing heart failure: report from the HOMAGE trial
Source: Hypertens Res. 2024 Sep 6;47(11):3225–36. doi: 10.1038/s41440-024-01843-z (PMC11534698; doi:10.1038/s41440-024-01843-z)
Supplement: Supplementary file 2 — List of HOMAGE Investigators [file 41440_2024_1843_MOESM2_ESM.doc]

**List of HOMAGE Investigators**

| **First name** | **Surname** |
| --- | --- |
| Fozia Z. | Ahmed |
| Kei | Asayama |
| Erwan | Bozec |
| Hans P. | Brunner La Rocca |
| Franco | Cosmi |
| Tim | Collier |
| Frank | Edelmann |
| Stephanie | Grojean |
| Mark | Hazebroek |
| Stephane | Heymans |
| Tine W. | Hansen |
| Javed | Khan |
| Begoñia | López |
| Roberto | Latini |
| Ken | McDonald |
| Gladys E. | Maestre |
| María U. | Moreno |
| Mamas A. | Mamas |
| Anne | Pizard |
| Burkert | Pieske |
| Philippe | Rouet |
| Lutgarde | Thijs |
